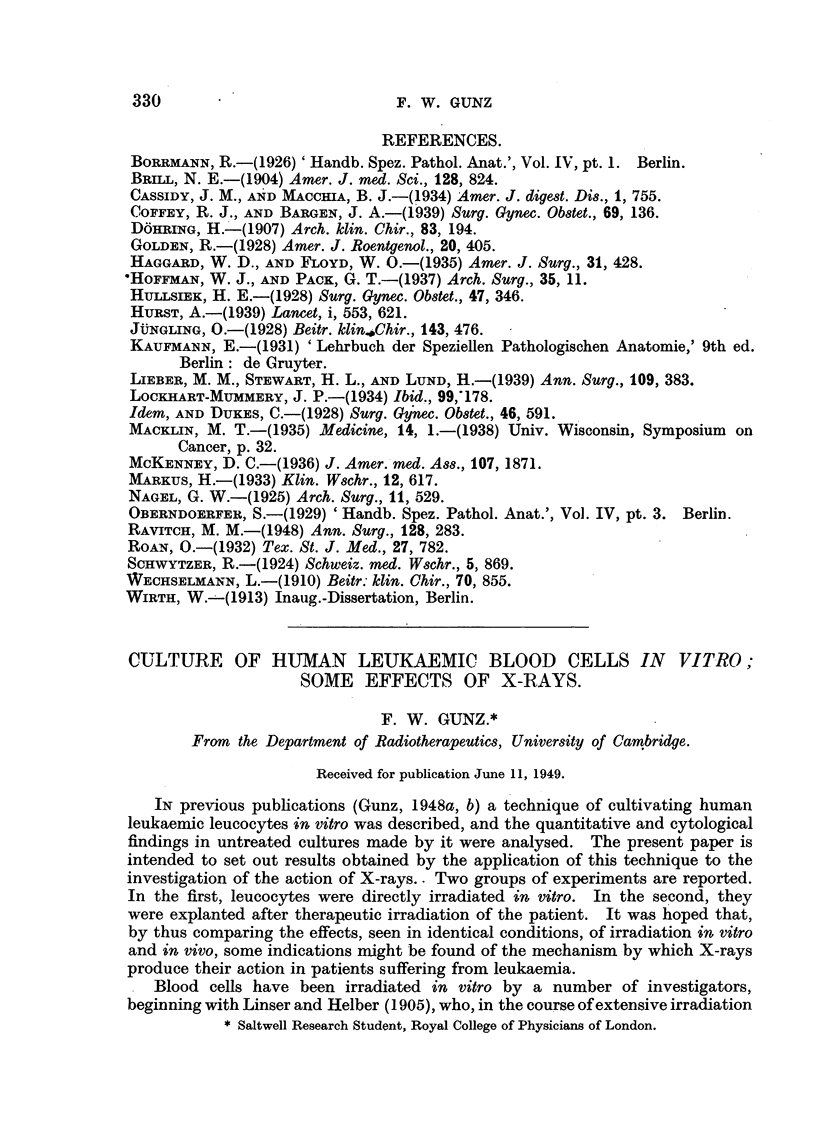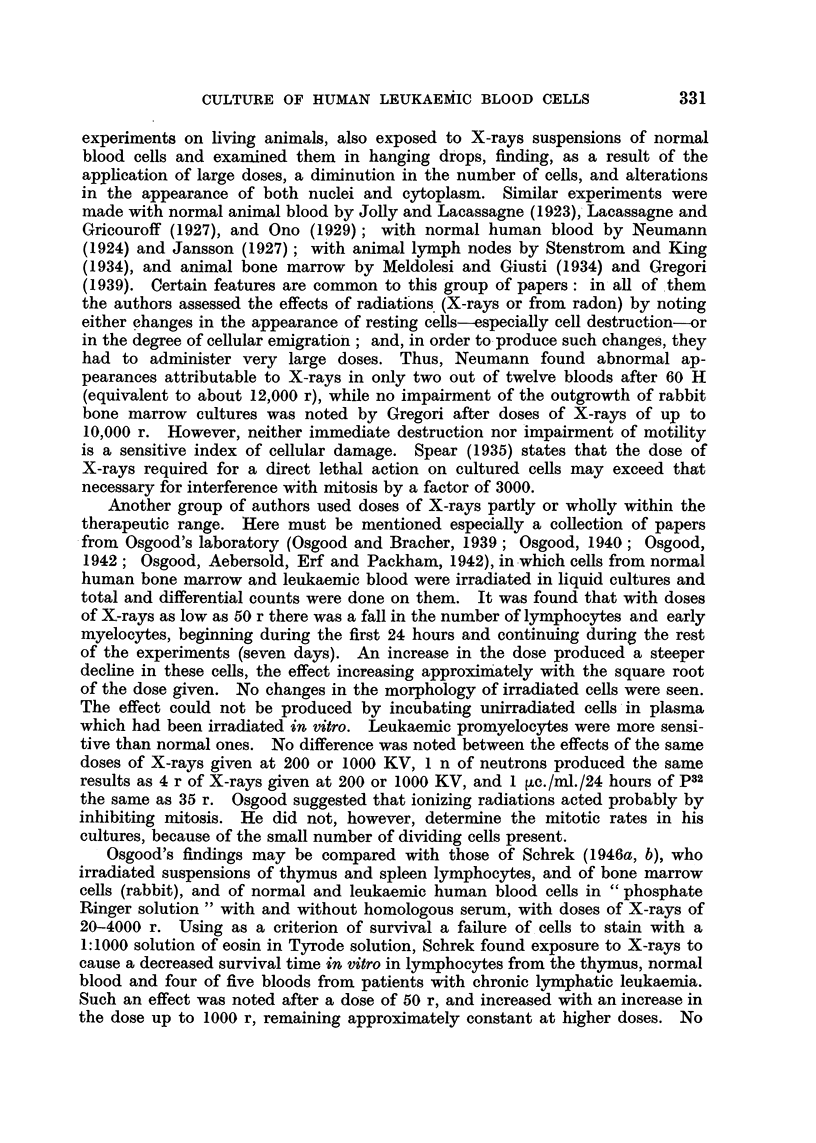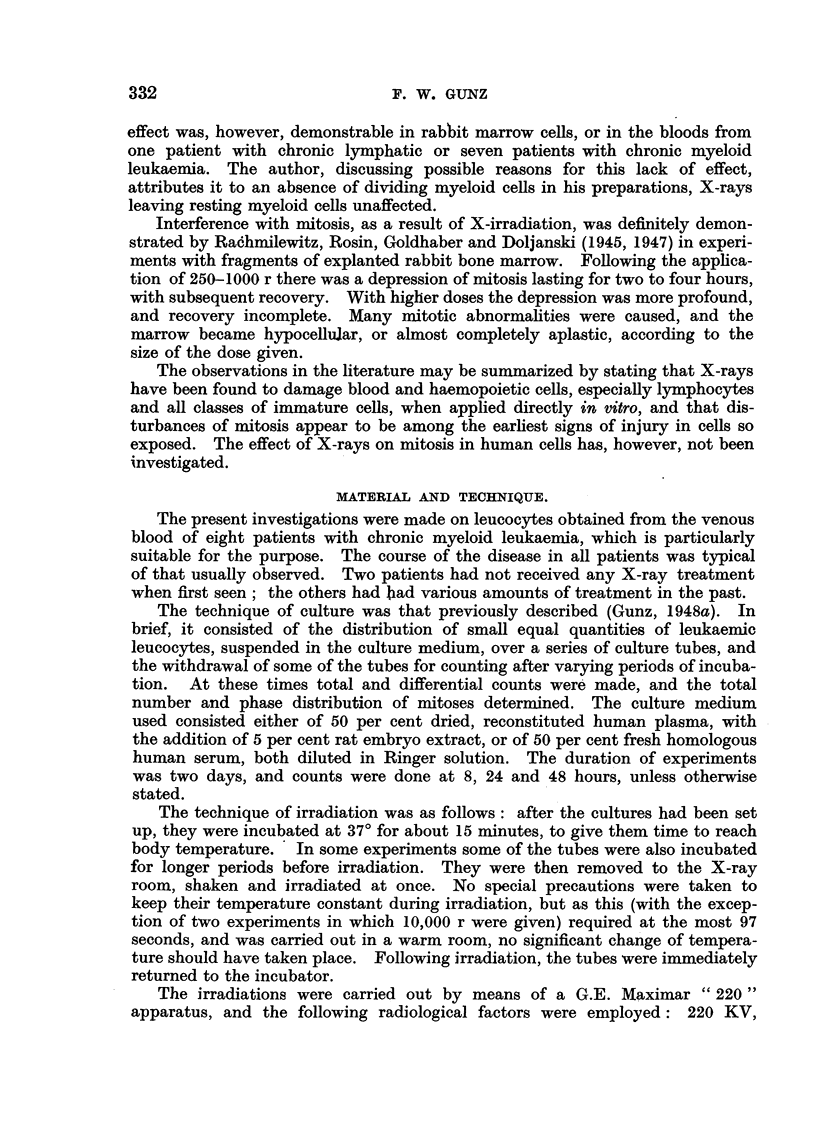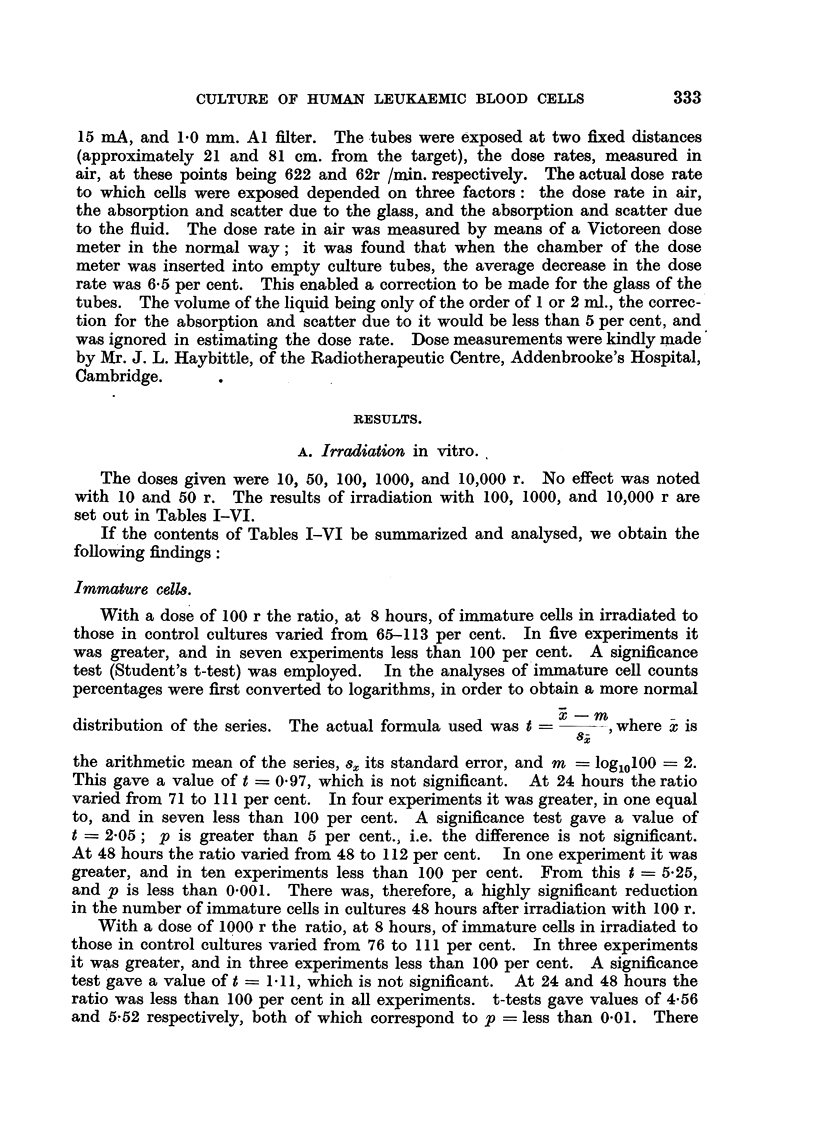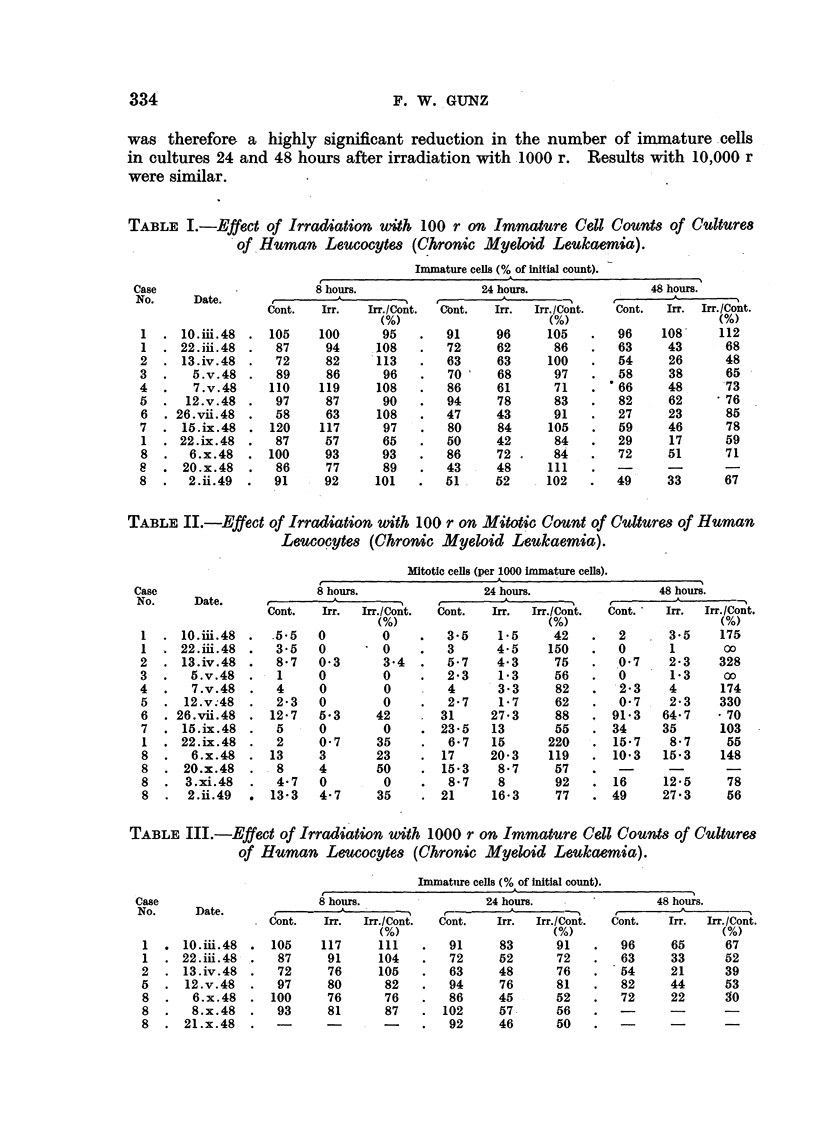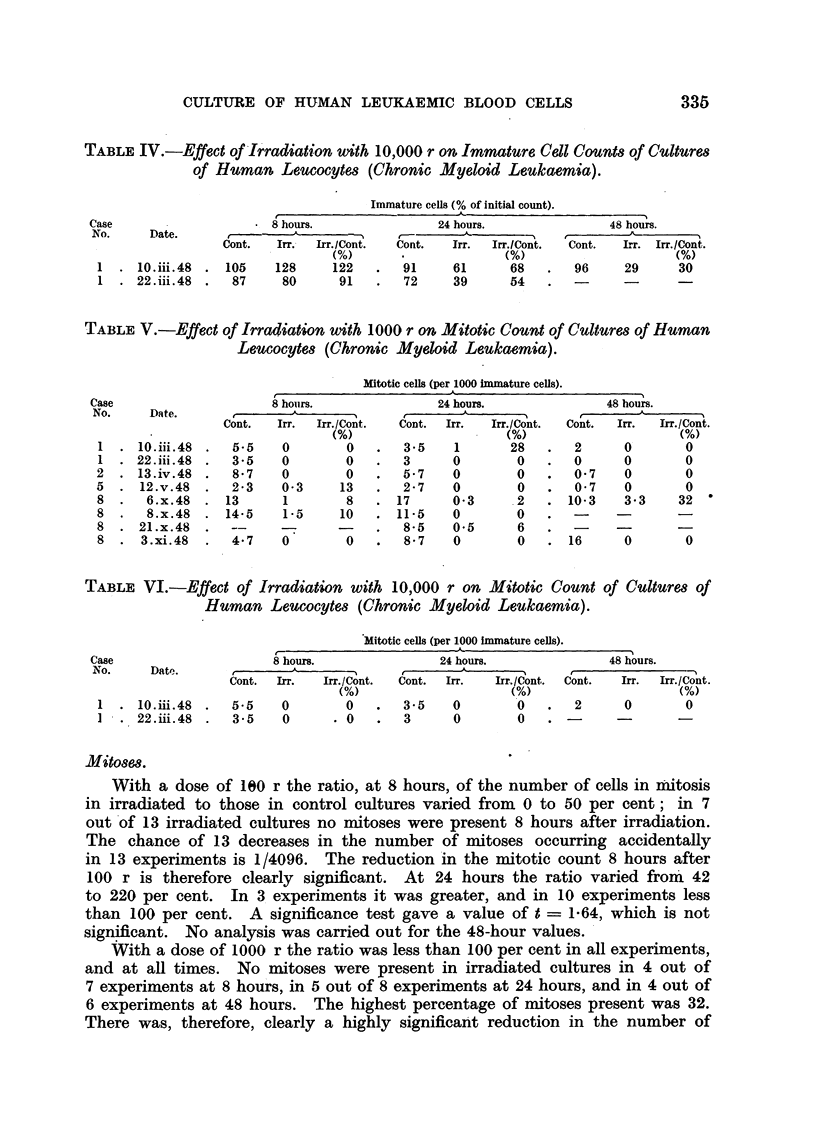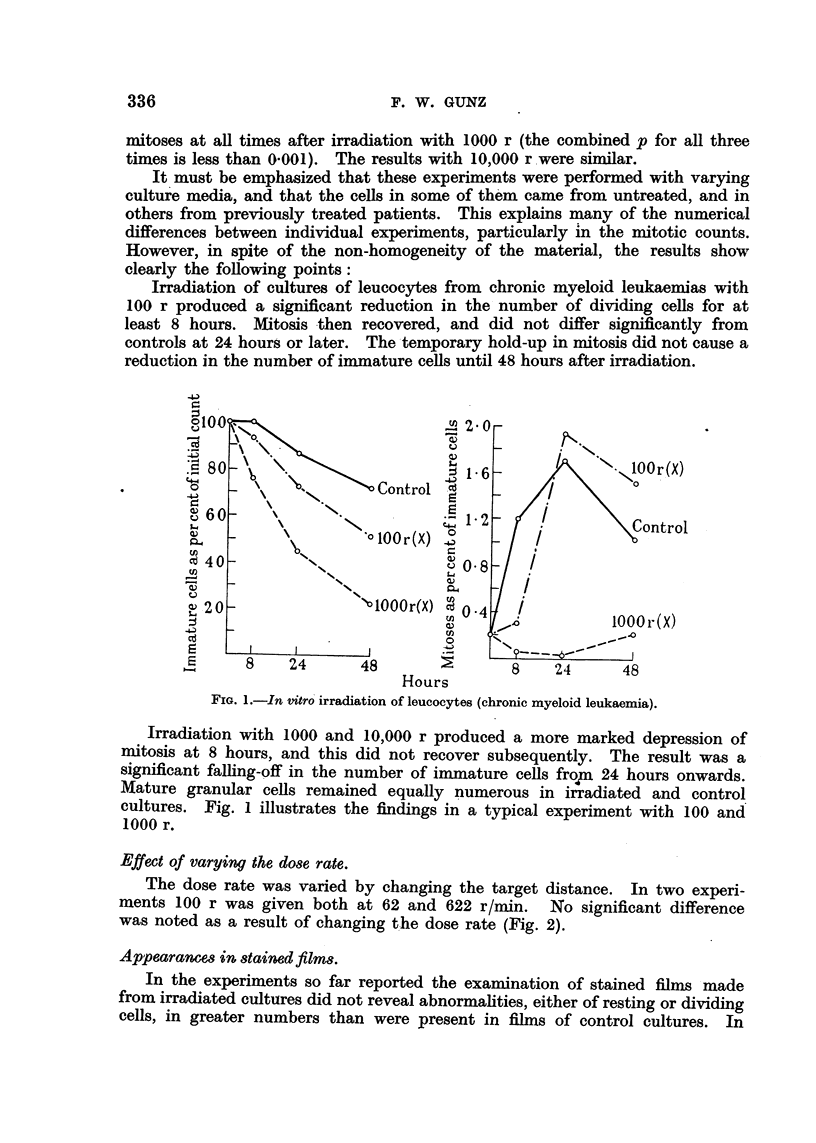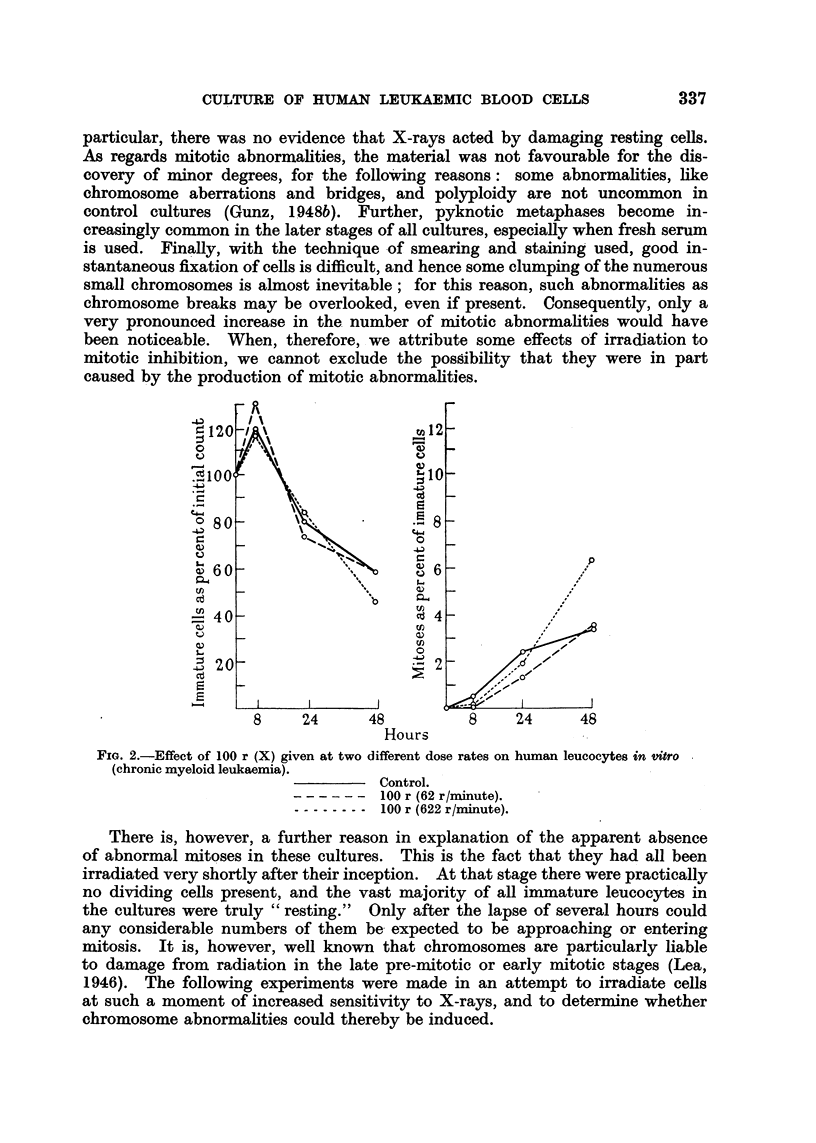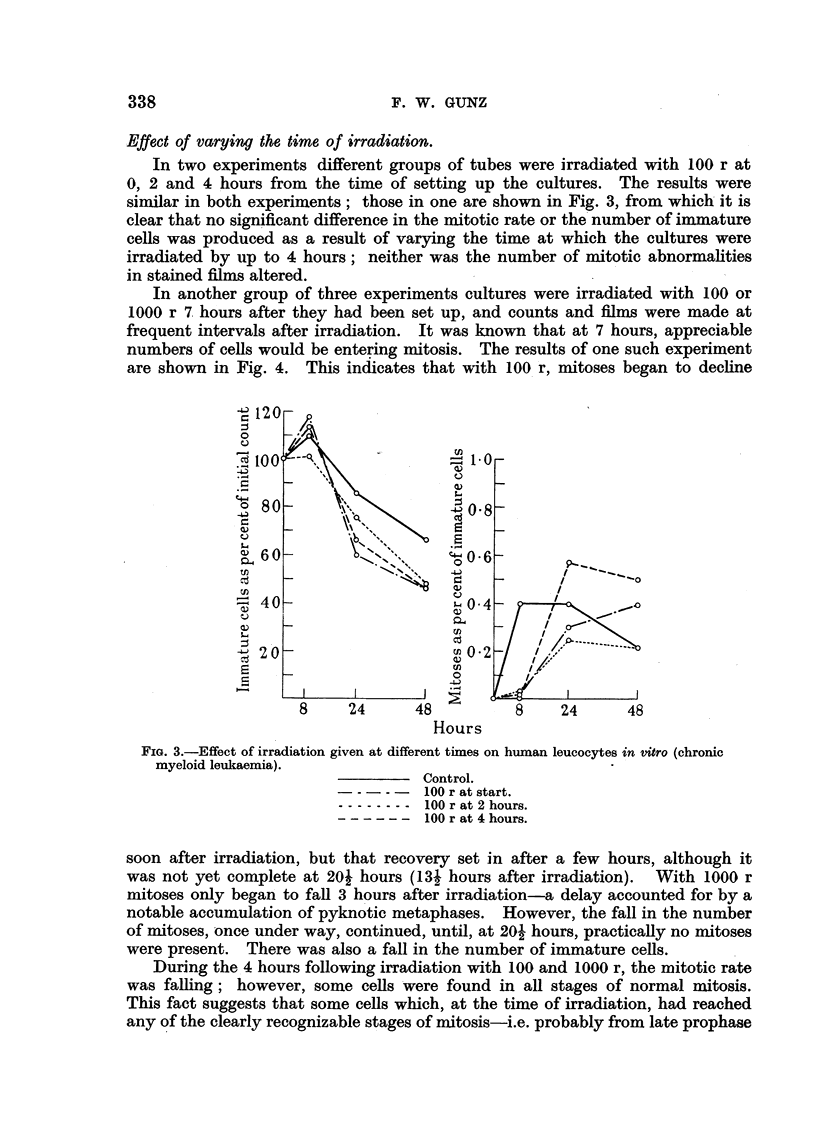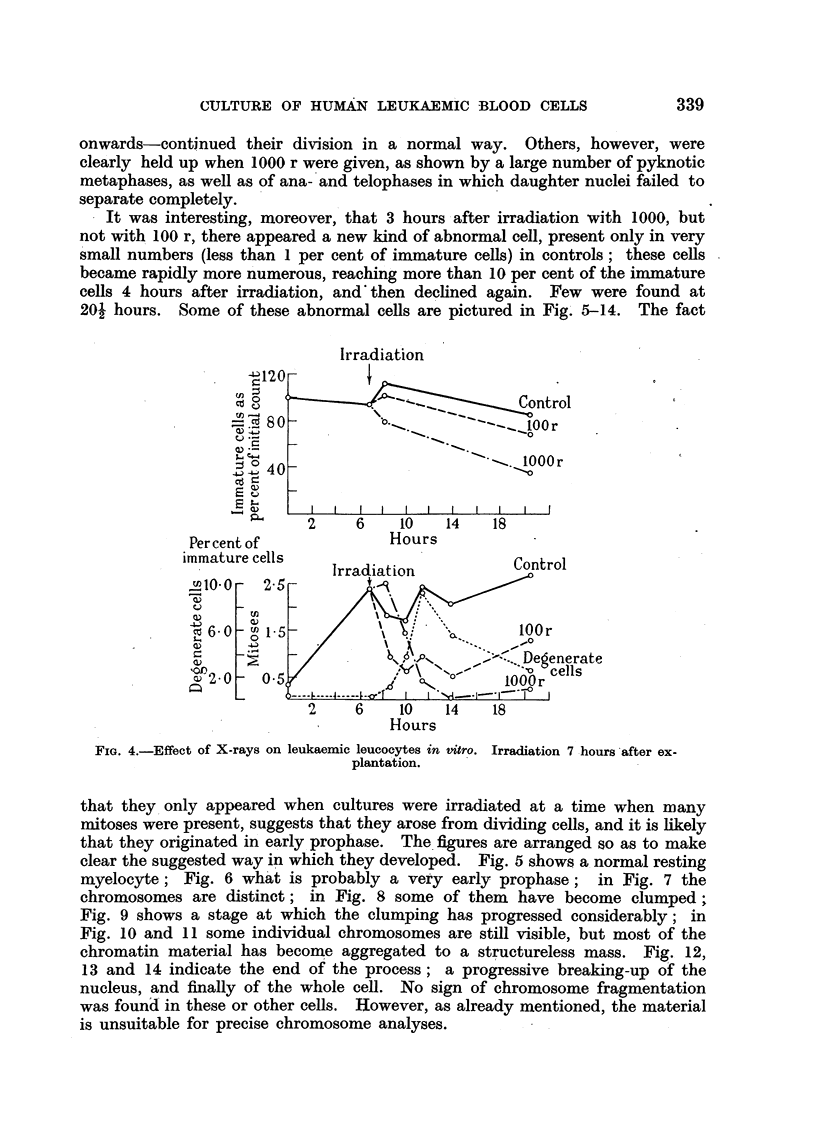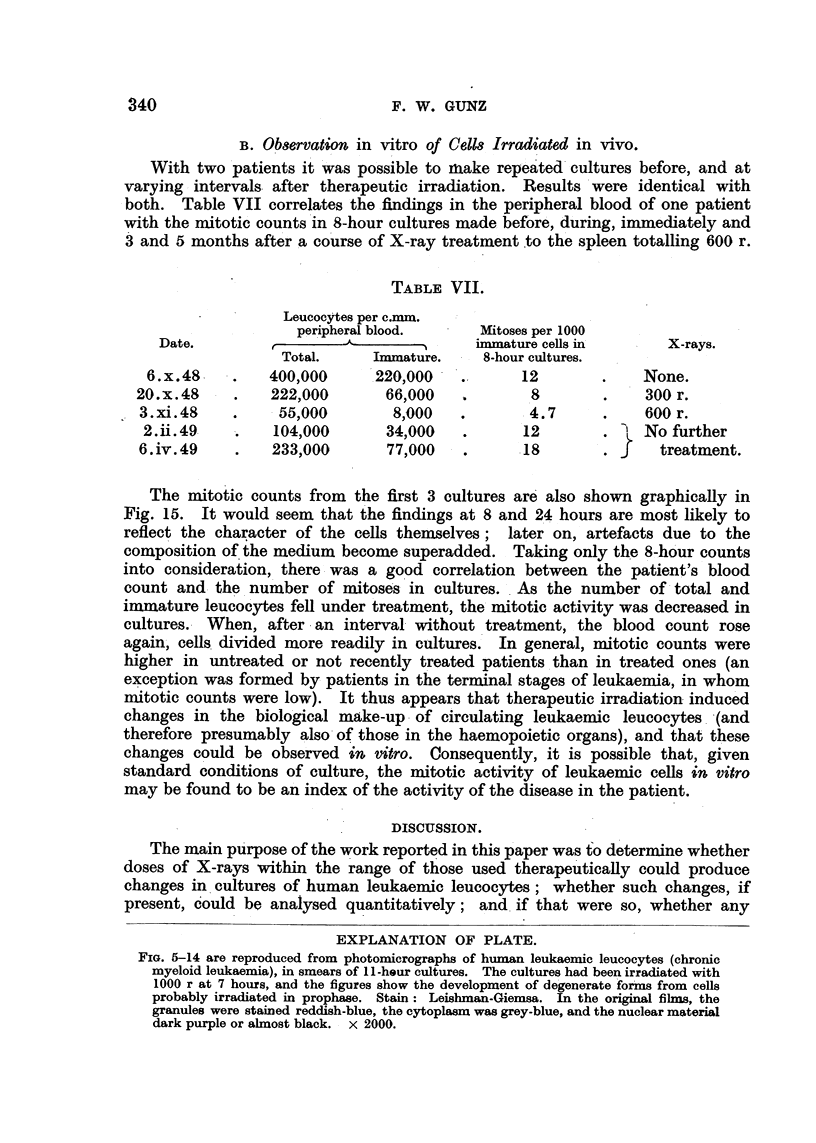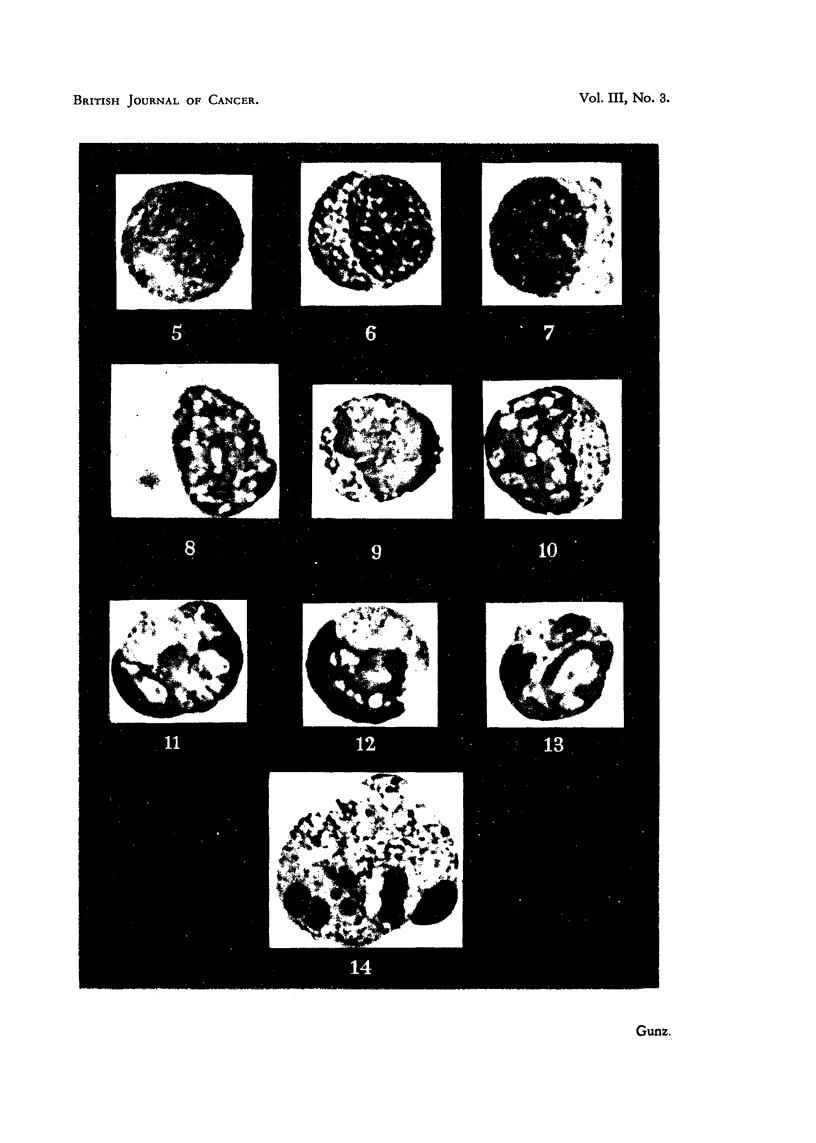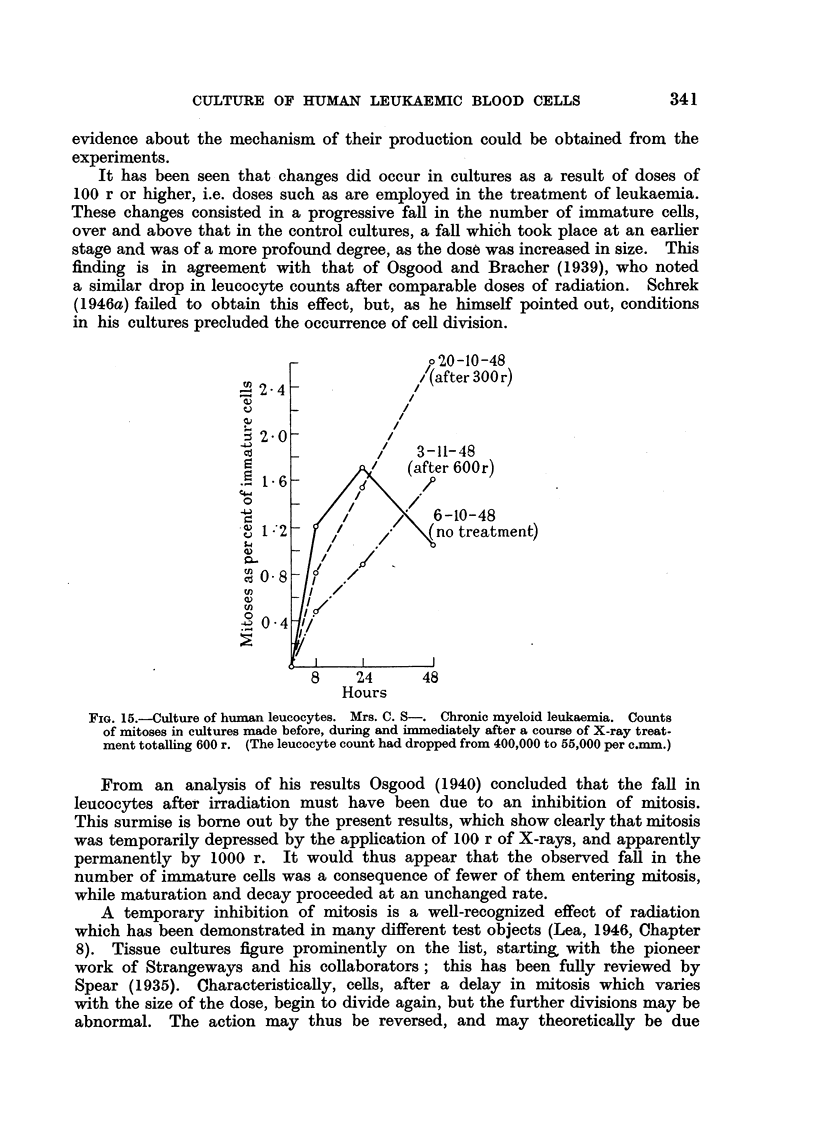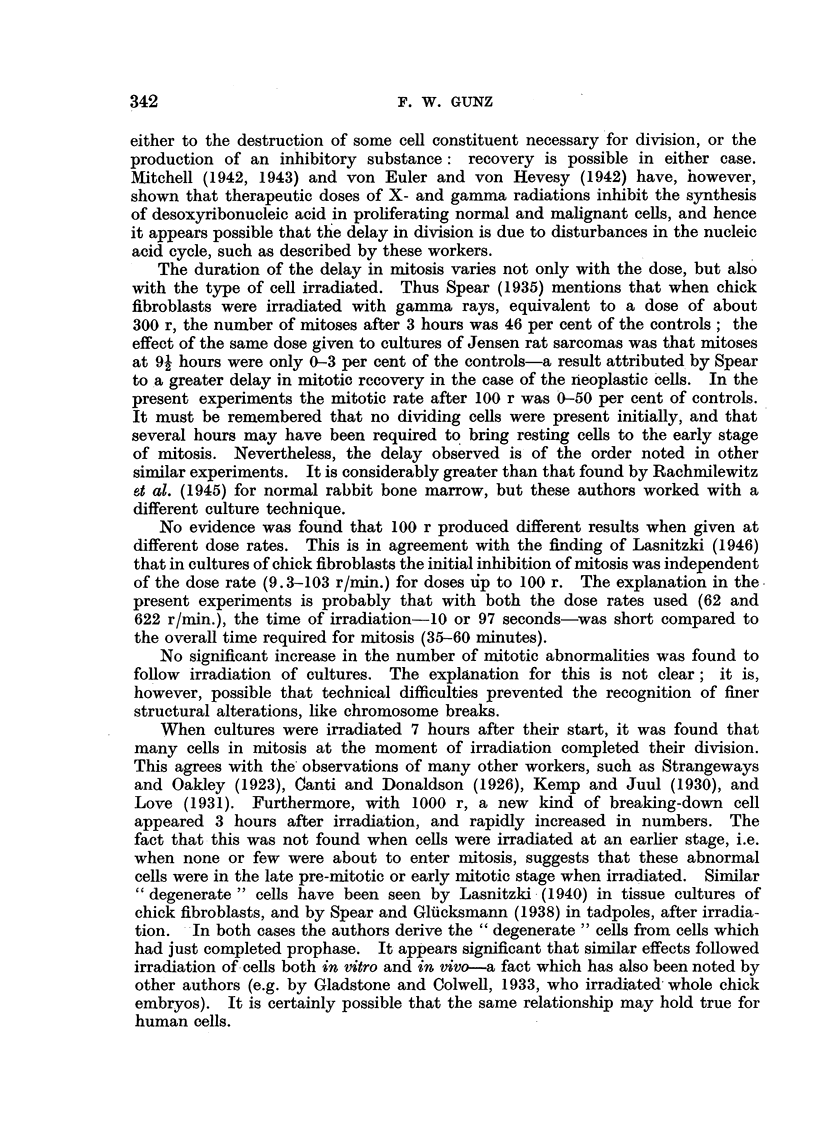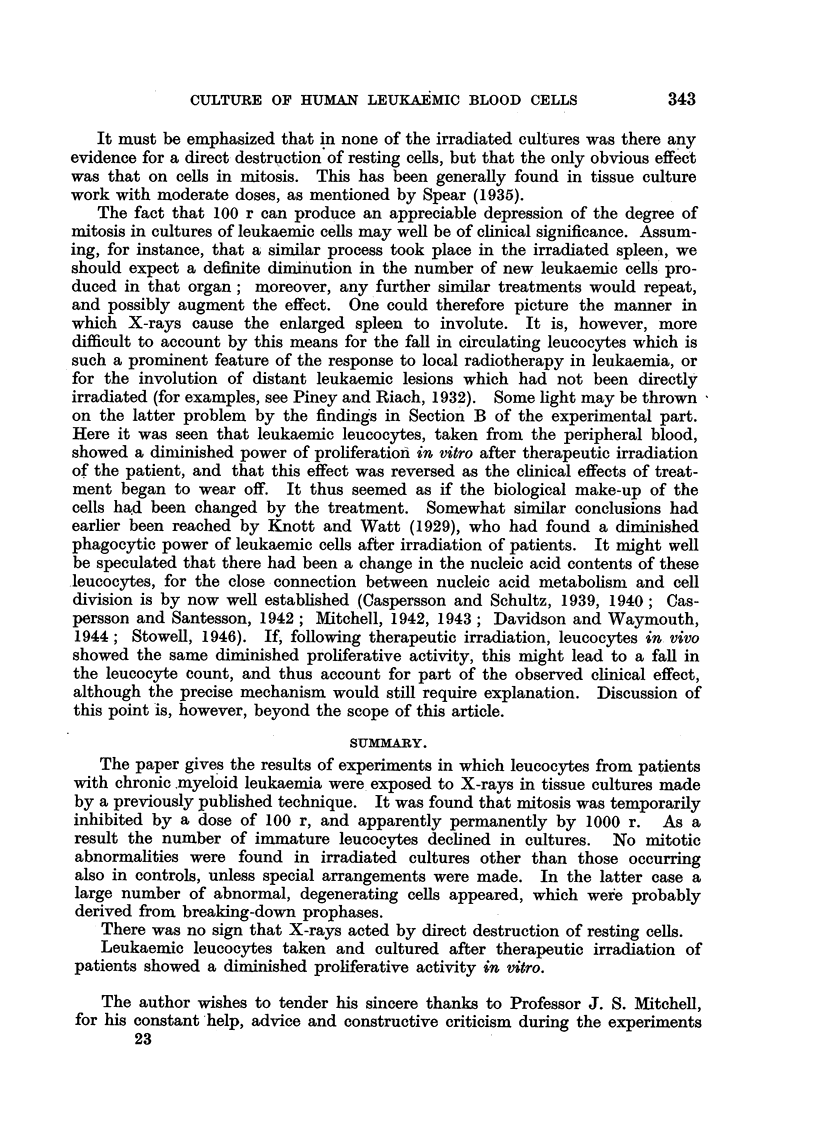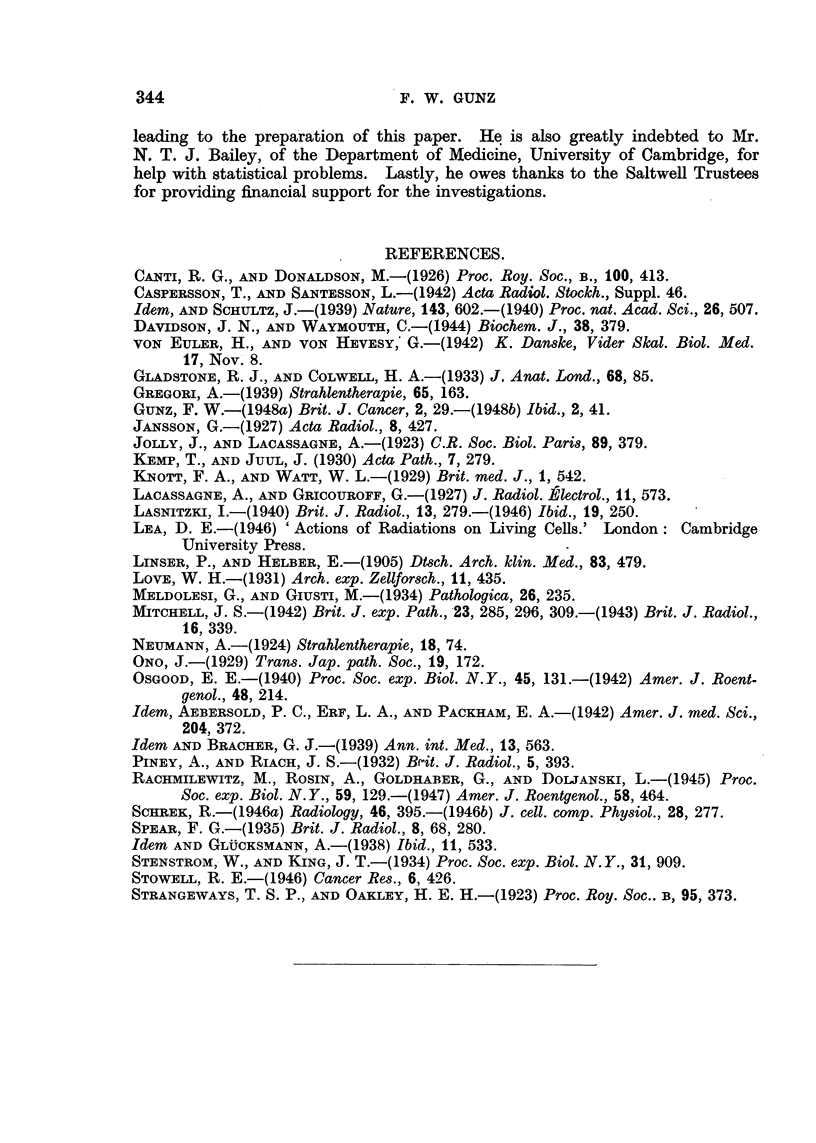# Culture of Human Leukaemic Blood Cells In Vitro; Some Effects of X-rays

**DOI:** 10.1038/bjc.1949.39

**Published:** 1949-09

**Authors:** F. W. Gunz

## Abstract

**Images:**


### CULTURE OF HUMAN LEUKAEMIC BLOOD CELLS *IN VITRO*; SOME EFFECTS OF X-RAYS.

F. W. GUNZ.*

*From the Department of Radiotherapeutics, University of Cambridge.*

Received for publication June 11, 1949.

IN previous publications (Gunz, 1948*a*, *b*) a technique of cultivating human leukaemic leucocytes *in vitro* was described, and the quantitative and cytological findings in untreated cultures made by it were analysed. The present paper is intended to set out results obtained by the application of this technique to the investigation of the action of X-rays. Two groups of experiments are reported. In the first, leucocytes were directly irradiated *in vitro*. In the second, they were explanted after therapeutic irradiation of the patient. It was hoped that, by thus comparing the effects, seen in identical conditions, of irradiation *in vitro* and *in vivo*, some indications might be found of the mechanism by which X-rays produce their action in patients suffering from leukaemia.

Blood cells have been irradiated *in vitro* by a number of investigators, beginning with Linser and Helber (1905), who, in the course of extensive irradiation

* Saltwell Research Student, Royal College of Physicians of London.

experiments on living animals, also exposed to X-rays suspensions of normal blood cells and examined them in hanging drops, finding, as a result of the application of large doses, a diminution in the number of cells, and alterations in the appearance of both nuclei and cytoplasm. Similar experiments were made with normal animal blood by Jolly and Lacassagne (1923), Lacassagne and Gricouroff (1927), and Ono (1929); with normal human blood by Neumann (1924) and Jansson (1927); with animal lymph nodes by Stenstrom and King (1934), and animal bone marrow by Meldolesi and Giusti (1934) and Gregori (1939). Certain features are common to this group of papers: in all of them the authors assessed the effects of radiations (X-rays or from radon) by noting either changes in the appearance of resting cells—especially cell destruction—or in the degree of cellular emigration; and, in order to produce such changes, they had to administer very large doses. Thus, Neumann found abnormal appearances attributable to X-rays in only two out of twelve bloods after 60 H (equivalent to about 12,000 r), while no impairment of the outgrowth of rabbit bone marrow cultures was noted by Gregori after doses of X-rays of up to 10,000 r. However, neither immediate destruction nor impairment of motility is a sensitive index of cellular damage. Spear (1935) states that the dose of X-rays required for a direct lethal action on cultured cells may exceed that necessary for interference with mitosis by a factor of 3000.

Another group of authors used doses of X-rays partly or wholly within the therapeutic range. Here must be mentioned especially a collection of papers from Osgood's laboratory (Osgood and Bracher, 1939; Osgood, 1940; Osgood, 1942; Osgood, Aebersold, Erf and Packham, 1942), in which cells from normal human bone marrow and leukaemic blood were irradiated in liquid cultures and total and differential counts were done on them. It was found that with doses of X-rays as low as 50 r there was a fall in the number of lymphocytes and early myelocytes, beginning during the first 24 hours and continuing during the rest of the experiments (seven days). An increase in the dose produced a steeper decline in these cells, the effect increasing approximately with the square root of the dose given. No changes in the morphology of irradiated cells were seen. The effect could not be produced by incubating unirradiated cells in plasma which had been irradiated *in vitro*. Leukaemic promyelocytes were more sensitive than normal ones. No difference was noted between the effects of the same doses of X-rays given at 200 or 1000 KV, 1 n of neutrons produced the same results as 4 r of X-rays given at 200 or 1000 KV, and 1 $\mu$c./ml./24 hours of $P^{32}$ the same as 35 r. Osgood suggested that ionizing radiations acted probably by inhibiting mitosis. He did not, however, determine the mitotic rates in his cultures, because of the small number of dividing cells present.

Osgood's findings may be compared with those of Schrek (1946*a*, *b*), who irradiated suspensions of thymus and spleen lymphocytes, and of bone marrow cells (rabbit), and of normal and leukaemic human blood cells in "phosphate Ringer solution" with and without homologous serum, with doses of X-rays of 20–4000 r. Using as a criterion of survival a failure of cells to stain with a 1:1000 solution of eosin in Tyrode solution, Schrek found exposure to X-rays to cause a decreased survival time *in vitro* in lymphocytes from the thymus, normal blood and four of five bloods from patients with chronic lymphatic leukaemia. Such an effect was noted after a dose of 50 r, and increased with an increase in the dose up to 1000 r, remaining approximately constant at higher doses. No effect was, however, demonstrable in rabbit marrow cells, or in the bloods from one patient with chronic lymphatic or seven patients with chronic myeloid leukaemia. The author, discussing possible reasons for this lack of effect, attributes it to an absence of dividing myeloid cells in his preparations, X-rays leaving resting myeloid cells unaffected.

Interference with mitosis, as a result of X-irradiation, was definitely demonstrated by Rachmilewitz, Rosin, Goldhaber and Doljanski (1945, 1947) in experiments with fragments of explanted rabbit bone marrow. Following the application of 250–1000 r there was a depression of mitosis lasting for two to four hours, with subsequent recovery. With higher doses the depression was more profound, and recovery incomplete. Many mitotic abnormalities were caused, and the marrow became hypocellular, or almost completely aplastic, according to the size of the dose given.

The observations in the literature may be summarized by stating that X-rays have been found to damage blood and haemopoietic cells, especially lymphocytes and all classes of immature cells, when applied directly *in vitro*, and that disturbances of mitosis appear to be among the earliest signs of injury in cells so exposed. The effect of X-rays on mitosis in human cells has, however, not been investigated.

### MATERIAL AND TECHNIQUE.

The present investigations were made on leucocytes obtained from the venous blood of eight patients with chronic myeloid leukaemia, which is particularly suitable for the purpose. The course of the disease in all patients was typical of that usually observed. Two patients had not received any X-ray treatment when first seen ; the others had had various amounts of treatment in the past.

The technique of culture was that previously described (Gunz, 1948*a*). In brief, it consisted of the distribution of small equal quantities of leukaemic leucocytes, suspended in the culture medium, over a series of culture tubes, and the withdrawal of some of the tubes for counting after varying periods of incubation. At these times total and differential counts were made, and the total number and phase distribution of mitoses determined. The culture medium used consisted either of 50 per cent dried, reconstituted human plasma, with the addition of 5 per cent rat embryo extract, or of 50 per cent fresh homologous human serum, both diluted in Ringer solution. The duration of experiments was two days, and counts were done at 8, 24 and 48 hours, unless otherwise stated.

The technique of irradiation was as follows : after the cultures had been set up, they were incubated at 37° for about 15 minutes, to give them time to reach body temperature. In some experiments some of the tubes were also incubated for longer periods before irradiation. They were then removed to the X-ray room, shaken and irradiated at once. No special precautions were taken to keep their temperature constant during irradiation, but as this (with the exception of two experiments in which 10,000 r were given) required at the most 97 seconds, and was carried out in a warm room, no significant change of temperature should have taken place. Following irradiation, the tubes were immediately returned to the incubator.

The irradiations were carried out by means of a G.E. Maximar " 220 " apparatus, and the following radiological factors were employed : 220 KV,

15 mA, and 1·0 mm. Al filter. The tubes were exposed at two fixed distances (approximately 21 and 81 cm. from the target), the dose rates, measured in air, at these points being 622 and 62r /min. respectively. The actual dose rate to which cells were exposed depended on three factors : the dose rate in air, the absorption and scatter due to the glass, and the absorption and scatter due to the fluid. The dose rate in air was measured by means of a Victoreen dose meter in the normal way ; it was found that when the chamber of the dose meter was inserted into empty culture tubes, the average decrease in the dose rate was 6·5 per cent. This enabled a correction to be made for the glass of the tubes. The volume of the liquid being only of the order of 1 or 2 ml., the correction for the absorption and scatter due to it would be less than 5 per cent, and was ignored in estimating the dose rate. Dose measurements were kindly made by Mr. J. L. Haybittle, of the Radiotherapeutic Centre, Addenbrooke's Hospital, Cambridge.

### RESULTS.

### A. *Irradiation* in vitro.

The doses given were 10, 50, 100, 1000, and 10,000 r. No effect was noted with 10 and 50 r. The results of irradiation with 100, 1000, and 10,000 r are set out in Tables I–VI.

If the contents of Tables I–VI be summarized and analysed, we obtain the following findings :

*Immature cells.*

With a dose of 100 r the ratio, at 8 hours, of immature cells in irradiated to those in control cultures varied from 65–113 per cent. In five experiments it was greater, and in seven experiments less than 100 per cent. A significance test (Student's t-test) was employed. In the analyses of immature cell counts percentages were first converted to logarithms, in order to obtain a more normal distribution of the series. The actual formula used was $t = \frac{\bar{x} - m}{s_{\bar{x}}}$, where $\bar{x}$ is the arithmetic mean of the series, $s_x$ its standard error, and $m = \log_{10}100 = 2$. This gave a value of $t = 0{\cdot}97$, which is not significant. At 24 hours the ratio varied from 71 to 111 per cent. In four experiments it was greater, in one equal to, and in seven less than 100 per cent. A significance test gave a value of $t = 2{\cdot}05$ ; $p$ is greater than 5 per cent., i.e. the difference is not significant. At 48 hours the ratio varied from 48 to 112 per cent. In one experiment it was greater, and in ten experiments less than 100 per cent. From this $t = 5{\cdot}25$, and $p$ is less than 0·001. There was, therefore, a highly significant reduction in the number of immature cells in cultures 48 hours after irradiation with 100 r.

With a dose of 1000 r the ratio, at 8 hours, of immature cells in irradiated to those in control cultures varied from 76 to 111 per cent. In three experiments it was greater, and in three experiments less than 100 per cent. A significance test gave a value of $t = 1{\cdot}11$, which is not significant. At 24 and 48 hours the ratio was less than 100 per cent in all experiments. t-tests gave values of 4·56 and 5·52 respectively, both of which correspond to $p =$ less than 0·01. There was therefore a highly significant reduction in the number of immature cells in cultures 24 and 48 hours after irradiation with 1000 r. Results with 10,000 r were similar.

TABLE I.—*Effect of Irradiation with* 100 *r on Immature Cell Counts of Cultures of Human Leucocytes (Chronic Myeloid Leukaemia).*

| Case No. | Date. | Immature cells (% of initial count). | | | | | | | | |
|---|---|---|---|---|---|---|---|---|---|---|
| | | 8 hours. | | | 24 hours. | | | 48 hours. | | |
| | | Cont. | Irr. | Irr./Cont. (%) | Cont. | Irr. | Irr./Cont. (%) | Cont. | Irr. | Irr./Cont. (%) |
| 1 | 10.iii.48 | 105 | 100 | 95 | 91 | 96 | 105 | 96 | 108 | 112 |
| 1 | 22.iii.48 | 87 | 94 | 108 | 72 | 62 | 86 | 63 | 43 | 68 |
| 2 | 13.iv.48 | 72 | 82 | 113 | 63 | 63 | 100 | 54 | 26 | 48 |
| 3 | 5.v.48 | 89 | 86 | 96 | 70 | 68 | 97 | 58 | 38 | 65 |
| 4 | 7.v.48 | 110 | 119 | 108 | 86 | 61 | 71 | 66 | 48 | 73 |
| 5 | 12.v.48 | 97 | 87 | 90 | 94 | 78 | 83 | 82 | 62 | 76 |
| 6 | 26.vii.48 | 58 | 63 | 108 | 47 | 43 | 91 | 27 | 23 | 85 |
| 7 | 15.ix.48 | 120 | 117 | 97 | 80 | 84 | 105 | 59 | 46 | 78 |
| 1 | 22.ix.48 | 87 | 57 | 65 | 50 | 42 | 84 | 29 | 17 | 59 |
| 8 | 6.x.48 | 100 | 93 | 93 | 86 | 72 | 84 | 72 | 51 | 71 |
| 8 | 20.x.48 | 86 | 77 | 89 | 43 | 48 | 111 | — | — | — |
| 8 | 2.ii.49 | 91 | 92 | 101 | 51 | 52 | 102 | 49 | 33 | 67 |

TABLE II.—*Effect of Irradiation with* 100 *r on Mitotic Count of Cultures of Human Leucocytes (Chronic Myeloid Leukaemia).*

| Case No. | Date. | Mitotic cells (per 1000 immature cells). | | | | | | | | |
|---|---|---|---|---|---|---|---|---|---|---|
| | | 8 hours. | | | 24 hours. | | | 48 hours. | | |
| | | Cont. | Irr. | Irr./Cont. (%) | Cont. | Irr. | Irr./Cont. (%) | Cont. | Irr. | Irr./Cont. (%) |
| 1 | 10.iii.48 | 5·5 | 0 | 0 | 3·5 | 1·5 | 42 | 2 | 3·5 | 175 |
| 1 | 22.iii.48 | 3·5 | 0 | 0 | 3 | 4·5 | 150 | 0 | 1 | ∞ |
| 2 | 13.iv.48 | 8·7 | 0·3 | 3·4 | 5·7 | 4·3 | 75 | 0·7 | 2·3 | 328 |
| 3 | 5.v.48 | 1 | 0 | 0 | 2·3 | 1·3 | 56 | 0 | 1·3 | ∞ |
| 4 | 7.v.48 | 4 | 0 | 0 | 4 | 3·3 | 82 | 2·3 | 4 | 174 |
| 5 | 12.v.48 | 2·3 | 0 | 0 | 2·7 | 1·7 | 62 | 0·7 | 2·3 | 330 |
| 6 | 26.vii.48 | 12·7 | 5·3 | 42 | 31 | 27·3 | 88 | 91·3 | 64·7 | 70 |
| 7 | 15.ix.48 | 5 | 0 | 0 | 23·5 | 13 | 55 | 34 | 35 | 103 |
| 1 | 22.ix.48 | 2 | 0·7 | 35 | 6·7 | 15 | 220 | 15·7 | 8·7 | 55 |
| 8 | 6.x.48 | 13 | 3 | 23 | 17 | 20·3 | 119 | 10·3 | 15·3 | 148 |
| 8 | 20.x.48 | 8 | 4 | 50 | 15·3 | 8·7 | 57 | — | — | — |
| 8 | 3.xi.48 | 4·7 | 0 | 0 | 8·7 | 8 | 92 | 16 | 12·5 | 78 |
| 8 | 2.ii.49 | 13·3 | 4·7 | 35 | 21 | 16·3 | 77 | 49 | 27·3 | 56 |

TABLE III.—*Effect of Irradiation with* 1000 *r on Immature Cell Counts of Cultures of Human Leucocytes (Chronic Myeloid Leukaemia).*

| Case No. | Date. | Immature cells (% of initial count). | | | | | | | | |
|---|---|---|---|---|---|---|---|---|---|---|
| | | 8 hours. | | | 24 hours. | | | 48 hours. | | |
| | | Cont. | Irr. | Irr./Cont. (%) | Cont. | Irr. | Irr./Cont. (%) | Cont. | Irr. | Irr./Cont. (%) |
| 1 | 10.iii.48 | 105 | 117 | 111 | 91 | 83 | 91 | 96 | 65 | 67 |
| 1 | 22.iii.48 | 87 | 91 | 104 | 72 | 52 | 72 | 63 | 33 | 52 |
| 2 | 13.iv.48 | 72 | 76 | 105 | 63 | 48 | 76 | 54 | 21 | 39 |
| 5 | 12.v.48 | 97 | 80 | 82 | 94 | 76 | 81 | 82 | 44 | 53 |
| 8 | 6.x.48 | 100 | 76 | 76 | 86 | 45 | 52 | 72 | 22 | 30 |
| 8 | 8.x.48 | 93 | 81 | 87 | 102 | 57 | 56 | — | — | — |
| 8 | 21.x.48 | — | — | — | 92 | 46 | 50 | — | — | — |

TABLE IV.—*Effect of Irradiation with* 10,000 *r on Immature Cell Counts of Cultures of Human Leucocytes (Chronic Myeloid Leukaemia).*

| Case No. | Date. | Immature cells (% of initial count). 8 hours. Cont. | Irr. | Irr./Cont. (%) | 24 hours. Cont. | Irr. | Irr./Cont. (%) | 48 hours. Cont. | Irr. | Irr./Cont. (%) |
|---|---|---|---|---|---|---|---|---|---|---|
| 1 | 10.iii.48 | 105 | 128 | 122 | 91 | 61 | 68 | 96 | 29 | 30 |
| 1 | 22.iii.48 | 87 | 80 | 91 | 72 | 39 | 54 | — | — | — |

TABLE V.—*Effect of Irradiation with* 1000 *r on Mitotic Count of Cultures of Human Leucocytes (Chronic Myeloid Leukaemia).*

| Case No. | Date. | Mitotic cells (per 1000 immature cells). 8 hours. Cont. | Irr. | Irr./Cont. (%) | 24 hours. Cont. | Irr. | Irr./Cont. (%) | 48 hours. Cont. | Irr. | Irr./Cont. (%) |
|---|---|---|---|---|---|---|---|---|---|---|
| 1 | 10.iii.48 | 5·5 | 0 | 0 | 3·5 | 1 | 28 | 2 | 0 | 0 |
| 1 | 22.iii.48 | 3·5 | 0 | 0 | 3 | 0 | 0 | 0 | 0 | 0 |
| 2 | 13.iv.48 | 8·7 | 0 | 0 | 5·7 | 0 | 0 | 0·7 | 0 | 0 |
| 5 | 12.v.48 | 2·3 | 0·3 | 13 | 2·7 | 0 | 0 | 0·7 | 0 | 0 |
| 8 | 6.x.48 | 13 | 1 | 8 | 17 | 0·3 | 2 | 10·3 | 3·3 | 32 |
| 8 | 8.x.48 | 14·5 | 1·5 | 10 | 11·5 | 0 | 0 | — | — | — |
| 8 | 21.x.48 | — | — | — | 8·5 | 0·5 | 6 | — | — | — |
| 8 | 3.xi.48 | 4·7 | 0 | 0 | 8·7 | 0 | 0 | 16 | 0 | 0 |

TABLE VI.—*Effect of Irradiation with* 10,000 *r on Mitotic Count of Cultures of Human Leucocytes (Chronic Myeloid Leukaemia).*

| Case No. | Date. | Mitotic cells (per 1000 immature cells). 8 hours. Cont. | Irr. | Irr./Cont. (%) | 24 hours. Cont. | Irr. | Irr./Cont. (%) | 48 hours. Cont. | Irr. | Irr./Cont. (%) |
|---|---|---|---|---|---|---|---|---|---|---|
| 1 | 10.iii.48 | 5·5 | 0 | 0 | 3·5 | 0 | 0 | 2 | 0 | 0 |
| 1 | 22.iii.48 | 3·5 | 0 | 0 | 3 | 0 | 0 | — | — | — |

*Mitoses.*

With a dose of 100 r the ratio, at 8 hours, of the number of cells in mitosis in irradiated to those in control cultures varied from 0 to 50 per cent; in 7 out of 13 irradiated cultures no mitoses were present 8 hours after irradiation. The chance of 13 decreases in the number of mitoses occurring accidentally in 13 experiments is 1/4096. The reduction in the mitotic count 8 hours after 100 r is therefore clearly significant. At 24 hours the ratio varied from 42 to 220 per cent. In 3 experiments it was greater, and in 10 experiments less than 100 per cent. A significance test gave a value of $t = 1{\cdot}64$, which is not significant. No analysis was carried out for the 48-hour values.

With a dose of 1000 r the ratio was less than 100 per cent in all experiments, and at all times. No mitoses were present in irradiated cultures in 4 out of 7 experiments at 8 hours, in 5 out of 8 experiments at 24 hours, and in 4 out of 6 experiments at 48 hours. The highest percentage of mitoses present was 32. There was, therefore, clearly a highly significant reduction in the number of mitoses at all times after irradiation with 1000 r (the combined $p$ for all three times is less than 0·001). The results with 10,000 r were similar.

It must be emphasized that these experiments were performed with varying culture media, and that the cells in some of them came from untreated, and in others from previously treated patients. This explains many of the numerical differences between individual experiments, particularly in the mitotic counts. However, in spite of the non-homogeneity of the material, the results show clearly the following points :

Irradiation of cultures of leucocytes from chronic myeloid leukaemias with 100 r produced a significant reduction in the number of dividing cells for at least 8 hours. Mitosis then recovered, and did not differ significantly from controls at 24 hours or later. The temporary hold-up in mitosis did not cause a reduction in the number of immature cells until 48 hours after irradiation.

FIG. 1.—*In vitro* irradiation of leucocytes (chronic myeloid leukaemia).

Irradiation with 1000 and 10,000 r produced a more marked depression of mitosis at 8 hours, and this did not recover subsequently. The result was a significant falling-off in the number of immature cells from 24 hours onwards. Mature granular cells remained equally numerous in irradiated and control cultures. Fig. 1 illustrates the findings in a typical experiment with 100 and 1000 r.

*Effect of varying the dose rate.*

The dose rate was varied by changing the target distance. In two experiments 100 r was given both at 62 and 622 r/min. No significant difference was noted as a result of changing the dose rate (Fig. 2).

*Appearances in stained films.*

In the experiments so far reported the examination of stained films made from irradiated cultures did not reveal abnormalities, either of resting or dividing cells, in greater numbers than were present in films of control cultures. In particular, there was no evidence that X-rays acted by damaging resting cells. As regards mitotic abnormalities, the material was not favourable for the discovery of minor degrees, for the following reasons: some abnormalities, like chromosome aberrations and bridges, and polyploidy are not uncommon in control cultures (Gunz, 1948*b*). Further, pyknotic metaphases become increasingly common in the later stages of all cultures, especially when fresh serum is used. Finally, with the technique of smearing and staining used, good instantaneous fixation of cells is difficult, and hence some clumping of the numerous small chromosomes is almost inevitable; for this reason, such abnormalities as chromosome breaks may be overlooked, even if present. Consequently, only a very pronounced increase in the number of mitotic abnormalities would have been noticeable. When, therefore, we attribute some effects of irradiation to mitotic inhibition, we cannot exclude the possibility that they were in part caused by the production of mitotic abnormalities.

FIG. 2.—Effect of 100 r (X) given at two different dose rates on human leucocytes *in vitro* (chronic myeloid leukaemia).

———— Control.
– – – – – – 100 r (62 r/minute).
· · · · · · · · 100 r (622 r/minute).

There is, however, a further reason in explanation of the apparent absence of abnormal mitoses in these cultures. This is the fact that they had all been irradiated very shortly after their inception. At that stage there were practically no dividing cells present, and the vast majority of all immature leucocytes in the cultures were truly "resting." Only after the lapse of several hours could any considerable numbers of them be expected to be approaching or entering mitosis. It is, however, well known that chromosomes are particularly liable to damage from radiation in the late pre-mitotic or early mitotic stages (Lea, 1946). The following experiments were made in an attempt to irradiate cells at such a moment of increased sensitivity to X-rays, and to determine whether chromosome abnormalities could thereby be induced.

*Effect of varying the time of irradiation.*

In two experiments different groups of tubes were irradiated with 100 r at 0, 2 and 4 hours from the time of setting up the cultures. The results were similar in both experiments ; those in one are shown in Fig. 3, from which it is clear that no significant difference in the mitotic rate or the number of immature cells was produced as a result of varying the time at which the cultures were irradiated by up to 4 hours ; neither was the number of mitotic abnormalities in stained films altered.

In another group of three experiments cultures were irradiated with 100 or 1000 r 7 hours after they had been set up, and counts and films were made at frequent intervals after irradiation. It was known that at 7 hours, appreciable numbers of cells would be entering mitosis. The results of one such experiment are shown in Fig. 4. This indicates that with 100 r, mitoses began to decline

FIG. 3.—Effect of irradiation given at different times on human leucocytes *in vitro* (chronic myeloid leukaemia).

———————— Control.
— · — · — 100 r at start.
· · · · · · · · 100 r at 2 hours.
– – – – – – 100 r at 4 hours.

soon after irradiation, but that recovery set in after a few hours, although it was not yet complete at 20½ hours (13½ hours after irradiation). With 1000 r mitoses only began to fall 3 hours after irradiation—a delay accounted for by a notable accumulation of pyknotic metaphases. However, the fall in the number of mitoses, once under way, continued, until, at 20½ hours, practically no mitoses were present. There was also a fall in the number of immature cells.

During the 4 hours following irradiation with 100 and 1000 r, the mitotic rate was falling ; however, some cells were found in all stages of normal mitosis. This fact suggests that some cells which, at the time of irradiation, had reached any of the clearly recognizable stages of mitosis—i.e. probably from late prophase onwards—continued their division in a normal way. Others, however, were clearly held up when 1000 r were given, as shown by a large number of pyknotic metaphases, as well as of ana- and telophases in which daughter nuclei failed to separate completely.

It was interesting, moreover, that 3 hours after irradiation with 1000, but not with 100 r, there appeared a new kind of abnormal cell, present only in very small numbers (less than 1 per cent of immature cells) in controls; these cells became rapidly more numerous, reaching more than 10 per cent of the immature cells 4 hours after irradiation, and then declined again. Few were found at 20½ hours. Some of these abnormal cells are pictured in Fig. 5–14. The fact

FIG. 4.—Effect of X-rays on leukaemic leucocytes *in vitro*. Irradiation 7 hours after explantation.

that they only appeared when cultures were irradiated at a time when many mitoses were present, suggests that they arose from dividing cells, and it is likely that they originated in early prophase. The figures are arranged so as to make clear the suggested way in which they developed. Fig. 5 shows a normal resting myelocyte; Fig. 6 what is probably a very early prophase; in Fig. 7 the chromosomes are distinct; in Fig. 8 some of them have become clumped; Fig. 9 shows a stage at which the clumping has progressed considerably; in Fig. 10 and 11 some individual chromosomes are still visible, but most of the chromatin material has become aggregated to a structureless mass. Fig. 12, 13 and 14 indicate the end of the process; a progressive breaking-up of the nucleus, and finally of the whole cell. No sign of chromosome fragmentation was found in these or other cells. However, as already mentioned, the material is unsuitable for precise chromosome analyses.

B. *Observation* in vitro *of Cells Irradiated* in vivo.

With two patients it was possible to make repeated cultures before, and at varying intervals after therapeutic irradiation. Results were identical with both. Table VII correlates the findings in the peripheral blood of one patient with the mitotic counts in 8-hour cultures made before, during, immediately and 3 and 5 months after a course of X-ray treatment to the spleen totalling 600 r.

TABLE VII.

| Date. | Leucocytes per c.mm. peripheral blood. Total. | Immature. | Mitoses per 1000 immature cells in 8-hour cultures. | X-rays. |
|---|---|---|---|---|
| 6.x.48 | 400,000 | 220,000 | 12 | None. |
| 20.x.48 | 222,000 | 66,000 | 8 | 300 r. |
| 3.xi.48 | 55,000 | 8,000 | 4.7 | 600 r. |
| 2.ii.49 | 104,000 | 34,000 | 12 | No further treatment. |
| 6.iv.49 | 233,000 | 77,000 | 18 | |

The mitotic counts from the first 3 cultures are also shown graphically in Fig. 15. It would seem that the findings at 8 and 24 hours are most likely to reflect the character of the cells themselves ; later on, artefacts due to the composition of the medium become superadded. Taking only the 8-hour counts into consideration, there was a good correlation between the patient's blood count and the number of mitoses in cultures. As the number of total and immature leucocytes fell under treatment, the mitotic activity was decreased in cultures. When, after an interval without treatment, the blood count rose again, cells divided more readily in cultures. In general, mitotic counts were higher in untreated or not recently treated patients than in treated ones (an exception was formed by patients in the terminal stages of leukaemia, in whom mitotic counts were low). It thus appears that therapeutic irradiation induced changes in the biological make-up of circulating leukaemic leucocytes (and therefore presumably also of those in the haemopoietic organs), and that these changes could be observed *in vitro*. Consequently, it is possible that, given standard conditions of culture, the mitotic activity of leukaemic cells *in vitro* may be found to be an index of the activity of the disease in the patient.

### DISCUSSION.

The main purpose of the work reported in this paper was to determine whether doses of X-rays within the range of those used therapeutically could produce changes in cultures of human leukaemic leucocytes ; whether such changes, if present, could be analysed quantitatively ; and if that were so, whether any

---

EXPLANATION OF PLATE.

FIG. 5–14 are reproduced from photomicrographs of human leukaemic leucocytes (chronic myeloid leukaemia), in smears of 11-hour cultures. The cultures had been irradiated with 1000 r at 7 hours, and the figures show the development of degenerate forms from cells probably irradiated in prophase. Stain : Leishman-Giemsa. In the original films, the granules were stained reddish-blue, the cytoplasm was grey-blue, and the nuclear material dark purple or almost black. × 2000.

Gunz.

evidence about the mechanism of their production could be obtained from the experiments.

It has been seen that changes did occur in cultures as a result of doses of 100 r or higher, i.e. doses such as are employed in the treatment of leukaemia. These changes consisted in a progressive fall in the number of immature cells, over and above that in the control cultures, a fall which took place at an earlier stage and was of a more profound degree, as the dose was increased in size. This finding is in agreement with that of Osgood and Bracher (1939), who noted a similar drop in leucocyte counts after comparable doses of radiation. Schrek (1946*a*) failed to obtain this effect, but, as he himself pointed out, conditions in his cultures precluded the occurrence of cell division.

FIG. 15.—Culture of human leucocytes. Mrs. C. S—. Chronic myeloid leukaemia. Counts of mitoses in cultures made before, during and immediately after a course of X-ray treatment totalling 600 r. (The leucocyte count had dropped from 400,000 to 55,000 per c.mm.)

From an analysis of his results Osgood (1940) concluded that the fall in leucocytes after irradiation must have been due to an inhibition of mitosis. This surmise is borne out by the present results, which show clearly that mitosis was temporarily depressed by the application of 100 r of X-rays, and apparently permanently by 1000 r. It would thus appear that the observed fall in the number of immature cells was a consequence of fewer of them entering mitosis, while maturation and decay proceeded at an unchanged rate.

A temporary inhibition of mitosis is a well-recognized effect of radiation which has been demonstrated in many different test objects (Lea, 1946, Chapter 8). Tissue cultures figure prominently on the list, starting with the pioneer work of Strangeways and his collaborators; this has been fully reviewed by Spear (1935). Characteristically, cells, after a delay in mitosis which varies with the size of the dose, begin to divide again, but the further divisions may be abnormal. The action may thus be reversed, and may theoretically be due either to the destruction of some cell constituent necessary for division, or the production of an inhibitory substance: recovery is possible in either case. Mitchell (1942, 1943) and von Euler and von Hevesy (1942) have, however, shown that therapeutic doses of X- and gamma radiations inhibit the synthesis of desoxyribonucleic acid in proliferating normal and malignant cells, and hence it appears possible that the delay in division is due to disturbances in the nucleic acid cycle, such as described by these workers.

The duration of the delay in mitosis varies not only with the dose, but also with the type of cell irradiated. Thus Spear (1935) mentions that when chick fibroblasts were irradiated with gamma rays, equivalent to a dose of about 300 r, the number of mitoses after 3 hours was 46 per cent of the controls; the effect of the same dose given to cultures of Jensen rat sarcomas was that mitoses at 9½ hours were only 0–3 per cent of the controls—a result attributed by Spear to a greater delay in mitotic recovery in the case of the neoplastic cells. In the present experiments the mitotic rate after 100 r was 0–50 per cent of controls. It must be remembered that no dividing cells were present initially, and that several hours may have been required to bring resting cells to the early stage of mitosis. Nevertheless, the delay observed is of the order noted in other similar experiments. It is considerably greater than that found by Rachmilewitz *et al.* (1945) for normal rabbit bone marrow, but these authors worked with a different culture technique.

No evidence was found that 100 r produced different results when given at different dose rates. This is in agreement with the finding of Lasnitzki (1946) that in cultures of chick fibroblasts the initial inhibition of mitosis was independent of the dose rate (9.3–103 r/min.) for doses up to 100 r. The explanation in the present experiments is probably that with both the dose rates used (62 and 622 r/min.), the time of irradiation—10 or 97 seconds—was short compared to the overall time required for mitosis (35–60 minutes).

No significant increase in the number of mitotic abnormalities was found to follow irradiation of cultures. The explanation for this is not clear; it is, however, possible that technical difficulties prevented the recognition of finer structural alterations, like chromosome breaks.

When cultures were irradiated 7 hours after their start, it was found that many cells in mitosis at the moment of irradiation completed their division. This agrees with the observations of many other workers, such as Strangeways and Oakley (1923), Canti and Donaldson (1926), Kemp and Juul (1930), and Love (1931). Furthermore, with 1000 r, a new kind of breaking-down cell appeared 3 hours after irradiation, and rapidly increased in numbers. The fact that this was not found when cells were irradiated at an earlier stage, i.e. when none or few were about to enter mitosis, suggests that these abnormal cells were in the late pre-mitotic or early mitotic stage when irradiated. Similar "degenerate" cells have been seen by Lasnitzki (1940) in tissue cultures of chick fibroblasts, and by Spear and Glücksmann (1938) in tadpoles, after irradiation. In both cases the authors derive the "degenerate" cells from cells which had just completed prophase. It appears significant that similar effects followed irradiation of cells both *in vitro* and *in vivo*—a fact which has also been noted by other authors (e.g. by Gladstone and Colwell, 1933, who irradiated whole chick embryos). It is certainly possible that the same relationship may hold true for human cells.

It must be emphasized that in none of the irradiated cultures was there any evidence for a direct destruction of resting cells, but that the only obvious effect was that on cells in mitosis. This has been generally found in tissue culture work with moderate doses, as mentioned by Spear (1935).

The fact that 100 r can produce an appreciable depression of the degree of mitosis in cultures of leukaemic cells may well be of clinical significance. Assuming, for instance, that a similar process took place in the irradiated spleen, we should expect a definite diminution in the number of new leukaemic cells produced in that organ ; moreover, any further similar treatments would repeat, and possibly augment the effect. One could therefore picture the manner in which X-rays cause the enlarged spleen to involute. It is, however, more difficult to account by this means for the fall in circulating leucocytes which is such a prominent feature of the response to local radiotherapy in leukaemia, or for the involution of distant leukaemic lesions which had not been directly irradiated (for examples, see Piney and Riach, 1932). Some light may be thrown on the latter problem by the findings in Section B of the experimental part. Here it was seen that leukaemic leucocytes, taken from the peripheral blood, showed a diminished power of proliferation *in vitro* after therapeutic irradiation of the patient, and that this effect was reversed as the clinical effects of treatment began to wear off. It thus seemed as if the biological make-up of the cells had been changed by the treatment. Somewhat similar conclusions had earlier been reached by Knott and Watt (1929), who had found a diminished phagocytic power of leukaemic cells after irradiation of patients. It might well be speculated that there had been a change in the nucleic acid contents of these leucocytes, for the close connection between nucleic acid metabolism and cell division is by now well established (Caspersson and Schultz, 1939, 1940 ; Caspersson and Santesson, 1942 ; Mitchell, 1942, 1943 ; Davidson and Waymouth, 1944 ; Stowell, 1946). If, following therapeutic irradiation, leucocytes *in vivo* showed the same diminished proliferative activity, this might lead to a fall in the leucocyte count, and thus account for part of the observed clinical effect, although the precise mechanism would still require explanation. Discussion of this point is, however, beyond the scope of this article.

### SUMMARY.

The paper gives the results of experiments in which leucocytes from patients with chronic myeloid leukaemia were exposed to X-rays in tissue cultures made by a previously published technique. It was found that mitosis was temporarily inhibited by a dose of 100 r, and apparently permanently by 1000 r. As a result the number of immature leucocytes declined in cultures. No mitotic abnormalities were found in irradiated cultures other than those occurring also in controls, unless special arrangements were made. In the latter case a large number of abnormal, degenerating cells appeared, which were probably derived from breaking-down prophases.

There was no sign that X-rays acted by direct destruction of resting cells.

Leukaemic leucocytes taken and cultured after therapeutic irradiation of patients showed a diminished proliferative activity *in vitro*.

The author wishes to tender his sincere thanks to Professor J. S. Mitchell, for his constant help, advice and constructive criticism during the experiments

23

leading to the preparation of this paper. He is also greatly indebted to Mr. N. T. J. Bailey, of the Department of Medicine, University of Cambridge, for help with statistical problems. Lastly, he owes thanks to the Saltwell Trustees for providing financial support for the investigations.

### REFERENCES.

Canti, R. G., and Donaldson, M.—(1926) *Proc. Roy. Soc.*, B., **100**, 413.
Caspersson, T., and Santesson, L.—(1942) *Acta Radiol. Stockh.*, Suppl. 46.
*Idem*, and Schultz, J.—(1939) *Nature*, **143**, 602.—(1940) *Proc. nat. Acad. Sci.*, **26**, 507.
Davidson, J. N., and Waymouth, C.—(1944) *Biochem. J.*, **38**, 379.
von Euler, H., and von Hevesy, G.—(1942) *K. Danske, Vider Skal. Biol. Med.* **17**, Nov. 8.
Gladstone, R. J., and Colwell, H. A.—(1933) *J. Anat. Lond.*, **68**, 85.
Gregori, A.—(1939) *Strahlentherapie*, **65**, 163.
Gunz, F. W.—(1948*a*) *Brit. J. Cancer*, **2**, 29.—(1948*b*) *Ibid.*, **2**, 41.
Jansson, G.—(1927) *Acta Radiol.*, **8**, 427.
Jolly, J., and Lacassagne, A.—(1923) *C.R. Soc. Biol. Paris*, **89**, 379.
Kemp, T., and Juul, J. (1930) *Acta Path.*, **7**, 279.
Knott, F. A., and Watt, W. L.—(1929) *Brit. med. J.*, **1**, 542.
Lacassagne, A., and Gricouroff, G.—(1927) *J. Radiol. Électrol.*, **11**, 573.
Lasnitzki, I.—(1940) *Brit. J. Radiol.*, **13**, 279.—(1946) *Ibid.*, **19**, 250.
Lea, D. E.—(1946) 'Actions of Radiations on Living Cells.' London: Cambridge University Press.
Linser, P., and Helber, E.—(1905) *Dtsch. Arch. klin. Med.*, **83**, 479.
Love, W. H.—(1931) *Arch. exp. Zellforsch.*, **11**, 435.
Meldolesi, G., and Giusti, M.—(1934) *Pathologica*, **26**, 235.
Mitchell, J. S.—(1942) *Brit. J. exp. Path.*, **23**, 285, 296, 309.—(1943) *Brit. J. Radiol.*, **16**, 339.
Neumann, A.—(1924) *Strahlentherapie*, **18**, 74.
Ono, J.—(1929) *Trans. Jap. path. Soc.*, **19**, 172.
Osgood, E. E.—(1940) *Proc. Soc. exp. Biol. N.Y.*, **45**, 131.—(1942) *Amer. J. Roentgenol.*, **48**, 214.
*Idem*, Aebersold, P. C., Erf, L. A., and Packham, E. A.—(1942) *Amer. J. med. Sci.*, **204**, 372.
*Idem* and Bracher, G. J.—(1939) *Ann. int. Med.*, **13**, 563.
Piney, A., and Riach, J. S.—(1932) *Brit. J. Radiol.*, **5**, 393.
Rachmilewitz, M., Rosin, A., Goldhaber, G., and Doljanski, L.—(1945) *Proc. Soc. exp. Biol. N.Y.*, **59**, 129.—(1947) *Amer. J. Roentgenol.*, **58**, 464.
Schrek, R.—(1946*a*) *Radiology*, **46**, 395.—(1946*b*) *J. cell. comp. Physiol.*, **28**, 277.
Spear, F. G.—(1935) *Brit. J. Radiol.*, **8**, 68, 280.
*Idem* and Glücksmann, A.—(1938) *Ibid.*, **11**, 533.
Stenstrom, W., and King, J. T.—(1934) *Proc. Soc. exp. Biol. N.Y.*, **31**, 909.
Stowell, R. E.—(1946) *Cancer Res.*, **6**, 426.
Strangeways, T. S. P., and Oakley, H. E. H.—(1923) *Proc. Roy. Soc.*. B, **95**, 373.